# Quadruple Negative Metastatic Melanoma With Gain of SOX-11 Expression and TERT Mutation

**DOI:** 10.7759/cureus.66651

**Published:** 2024-08-11

**Authors:** Ekin Ozluk, Jennifer Lee, Eric X Wei

**Affiliations:** 1 Department of Pathology and Laboratory Medicine, Chobanian and Avedisian School of Medicine, Boston University, Boston, USA; 2 Department of Pathology, LSU (Louisiana State University) Health Shreveport, Shreveport, USA; 3 Department of Pathology, University of South Alabama, Mobile, USA

**Keywords:** tert mutation, gain of sox-11, spleen, melanoma, metastatic

## Abstract

Malignant melanoma is a common and aggressive skin cancer with a high incidence of metastases. Diagnosis is usually straightforward, based on a combination of histomorphology and immunohistochemistry. However, metastatic melanoma is notorious for its phenotypic diversity and loss of differentiation markers. Through recent developments in diagnostic immunohistochemistry and molecular pathology, several new markers are identified to be of use in confirming melanoma diagnosis, especially in undifferentiated and dedifferentiated cases. Here we report a challenging case of a 59-year-old male with splenic metastatic melanoma which revealed a loss of four diagnostic melanocytic markers including S100, SOX-10, HMB45, and MART-1, but a gain of SOX-11.

## Introduction

Loss of expression of one or more melanocytic markers is a well-known phenomenon in melanoma. Occasionally, melanoma may lose all of its routine diagnostic markers such as HMB45, MART-1, S100, and SOX-10 indicating dedifferentiation [1]. Although SOX-10 has a limited specificity, it is known to be highly sensitive for melanocytic neoplasms [2].

Undifferentiated or dedifferentiation processes may occur either in primary or metastatic melanoma. In rare circumstances, dedifferentiated melanoma can lose all differentiation markers, the so-called “vimentin only” type. While "undifferentiation" describes the loss of immunohistochemical markers and indicates a less differentiated status, “dedifferentiation” refers to a process that in addition to undifferentiation, includes the gaining of non-melanocytic features [3]. Melanomas may express mesenchymal and neuroectodermal components [4]. This is a potential pitfall for any pathologist.

Here, we present a case of dedifferentiated metastatic melanoma with the loss of four diagnostic melanocytic markers, but a gain of SOX-11.

## Case presentation

A 59-year-old Caucasian male presented with multiple splenic masses with 3 cm in greatest diameter. He had a left parotid mass one year prior to his latest arrival; the histopathological diagnosis was the intraparotid lymph node with metastatic malignant melanoma. The lesional cells showed strong positivity for S100, HMB45, and vimentin, with dedifferentiated foci that had a loss of S100 and HMB45. However, further clinical investigation failed to reveal the primary source. No BRAF V600E mutation was detected in the parotid mass. The treatments received were left total parotidectomy with immunotherapy/radiation, followed by Opdivo adjuvant therapy. The patient improved in the neck, but a follow-up PET scan showed avidity in the spleen.

**Figure 1:**
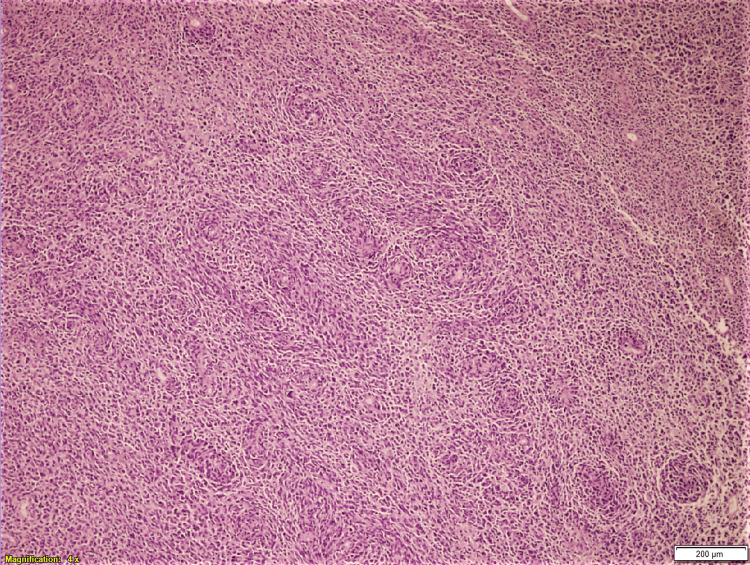
Metastatic melanoma, low power view Representative section shows sheets of discohesive neoplastic cells with pleomorphism (H&E, 4x magnification)

**Figure 2:**
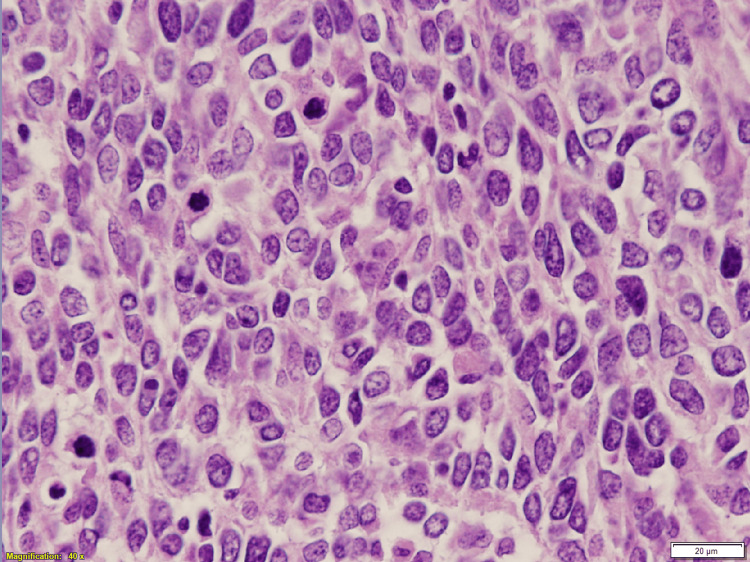
Metastatic melanoma, high power view High power shows discohesive, pleomorphic neoplastic cells with moderate amount of cytoplasm, irregular nuclear contour, clumped chromatin, and brisk mitotic activity (H&E, 40x magnification)

**Figure 3:**
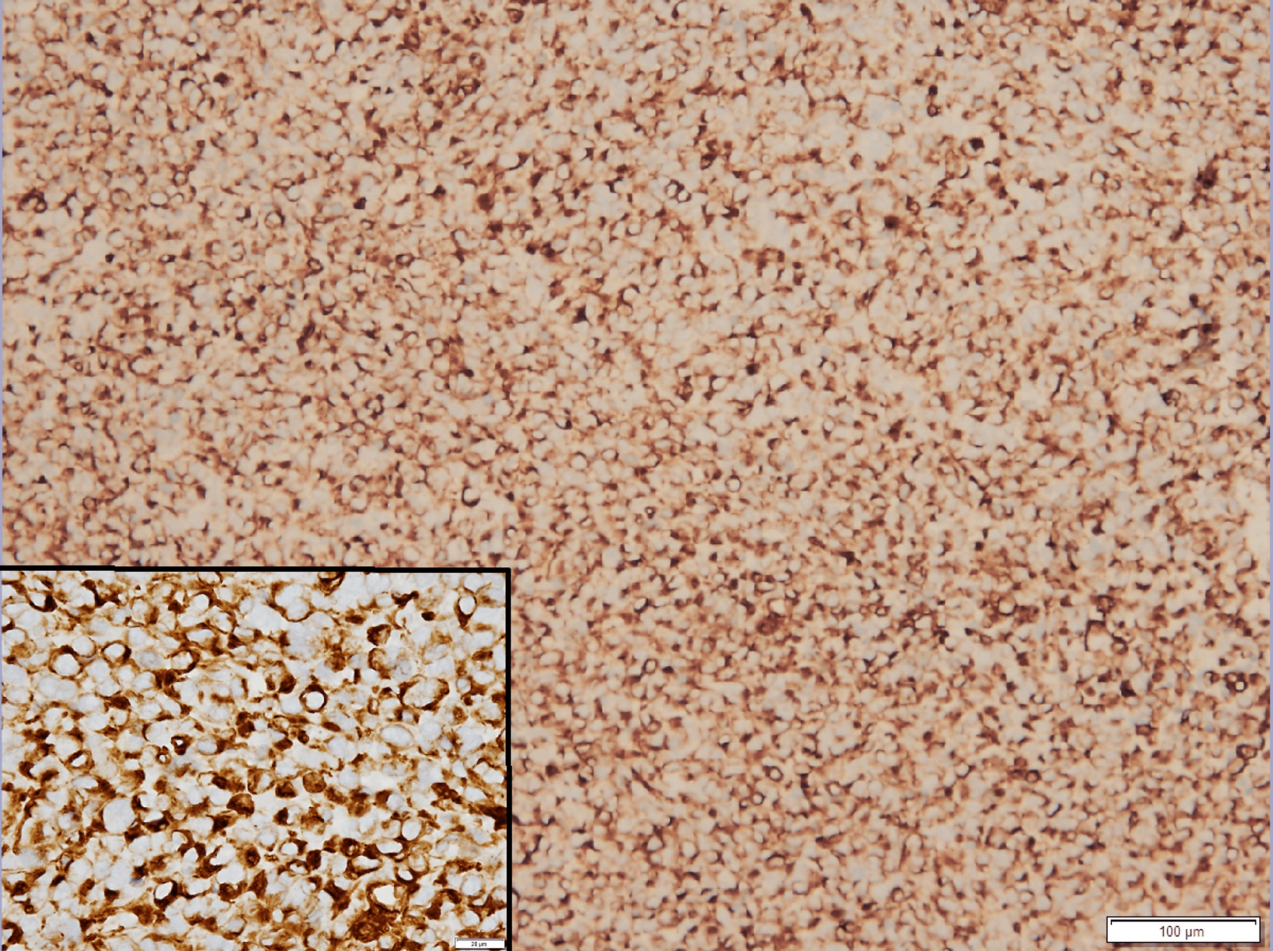
Neoplastic cells show cytoplasmic positivity of vimentin (Immunohistochemical stain, 10x, inset: 40x magnification)

**Figure 4:**
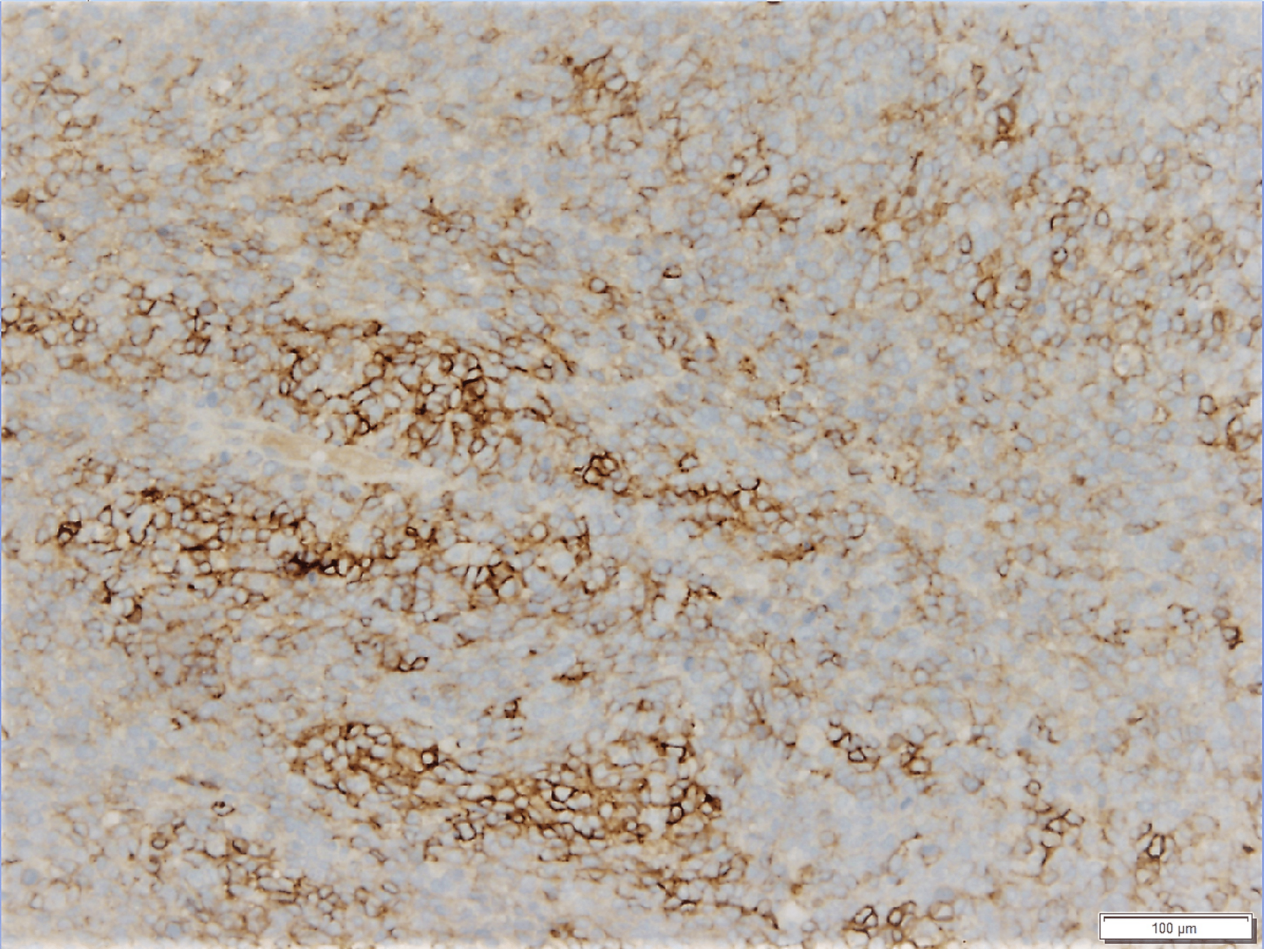
Some neoplastic cells show membranous positivity of CD56 (Immunohistochemical stain, 10x magnification)

**Figure 5:**
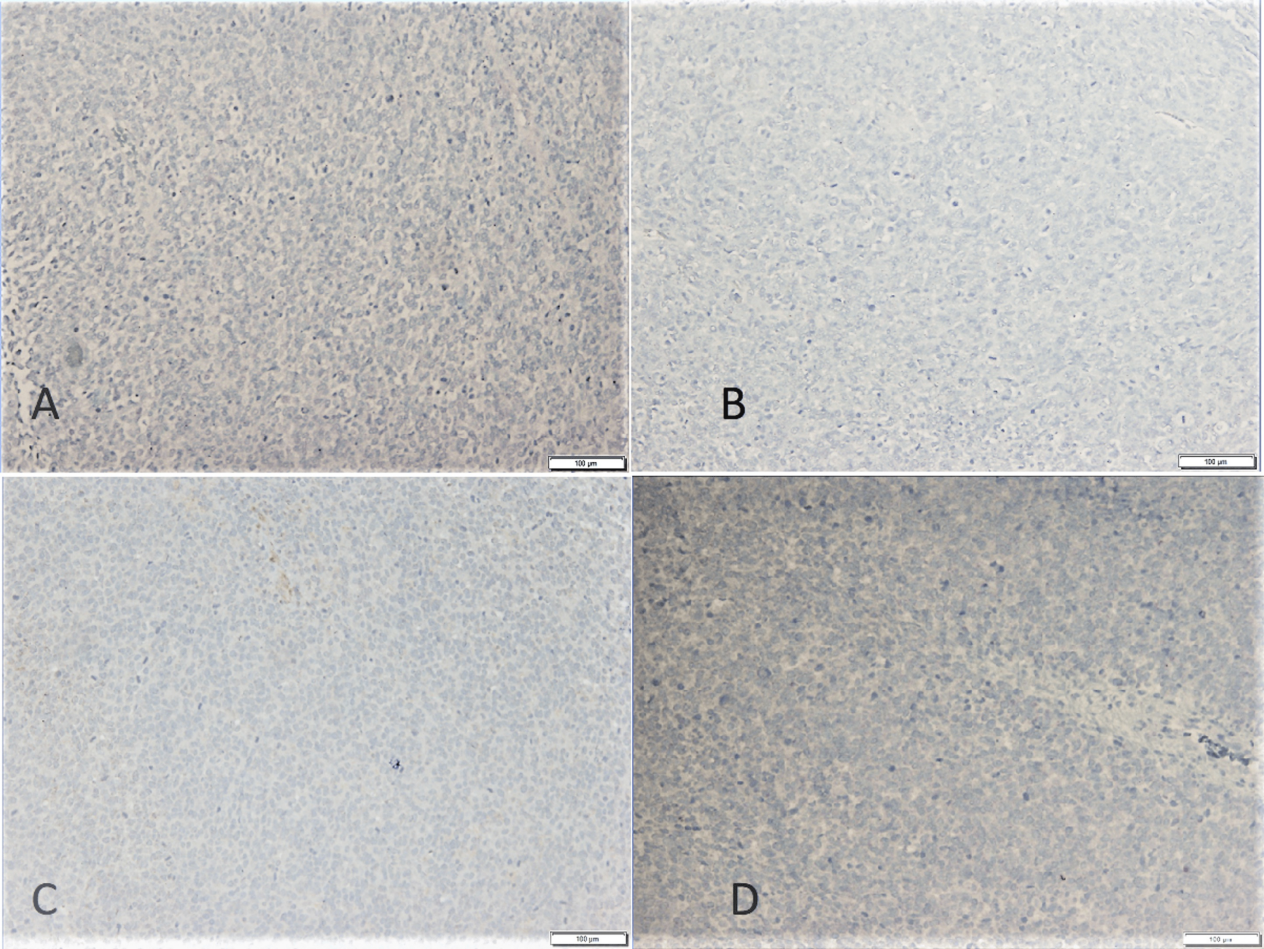
Melanocytic markers Neoplastic cells are negative for (A) S100, (B) HMB45, (C) SOX10, (D) MART1 (Immunohistochemical stain, 10x magnification)

**Figure 6:**
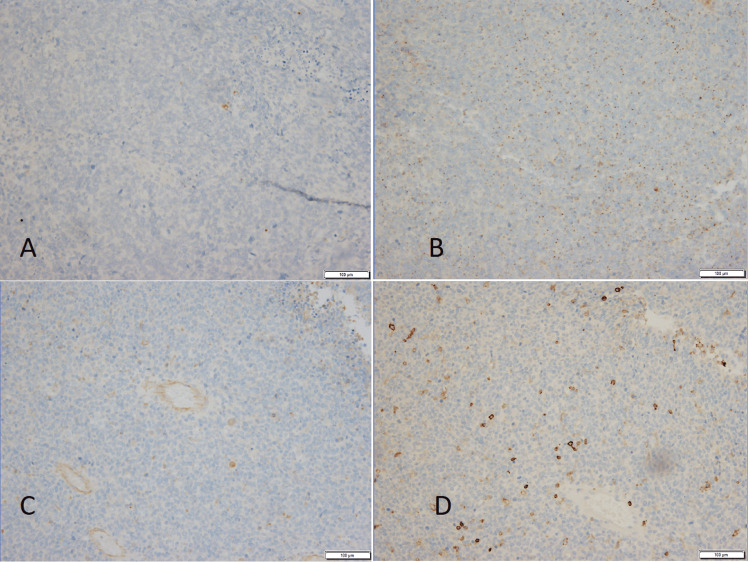
Immunohistochemical stain to exclude differential diagnoses. Neoplastic cells are negative for (A) Keratin AE1/AE3, (B) CD99, (C) PAX5, (D) LCA (10x magnification)

Splenectomy was performed to reveal an 842 gram spleen with a large hemorrhagic cystic space measuring 8 x 6 cm. Multiple gray-pink solid nodules were identified on cut surfaces, ranging from 1.5 cm to 3 cm in greatest diameter. Microscopically, the nodules were composed of sheets of neoplastic cells with marked pleomorphism, frequent mitoses, hemorrhage, and necrosis. They had clumped chromatin, irregular nuclear contours, and relatively abundant cytoplasm as seen in Figures 1-6.

**Figure 7:**
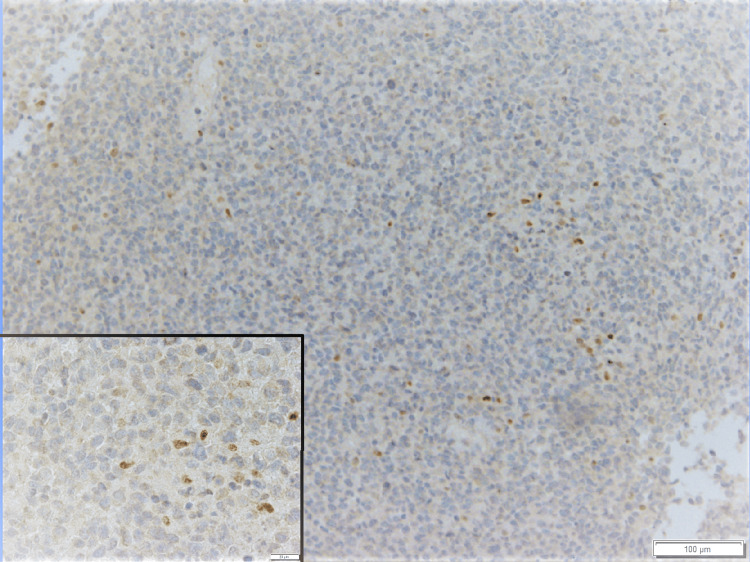
Few neoplastic cells show nuclear positivity of MiTF (Immunohistochemical stain, 10x, inset: 40x magnification) MiTF: microphthalmia transcription factor

**Figure 8:**
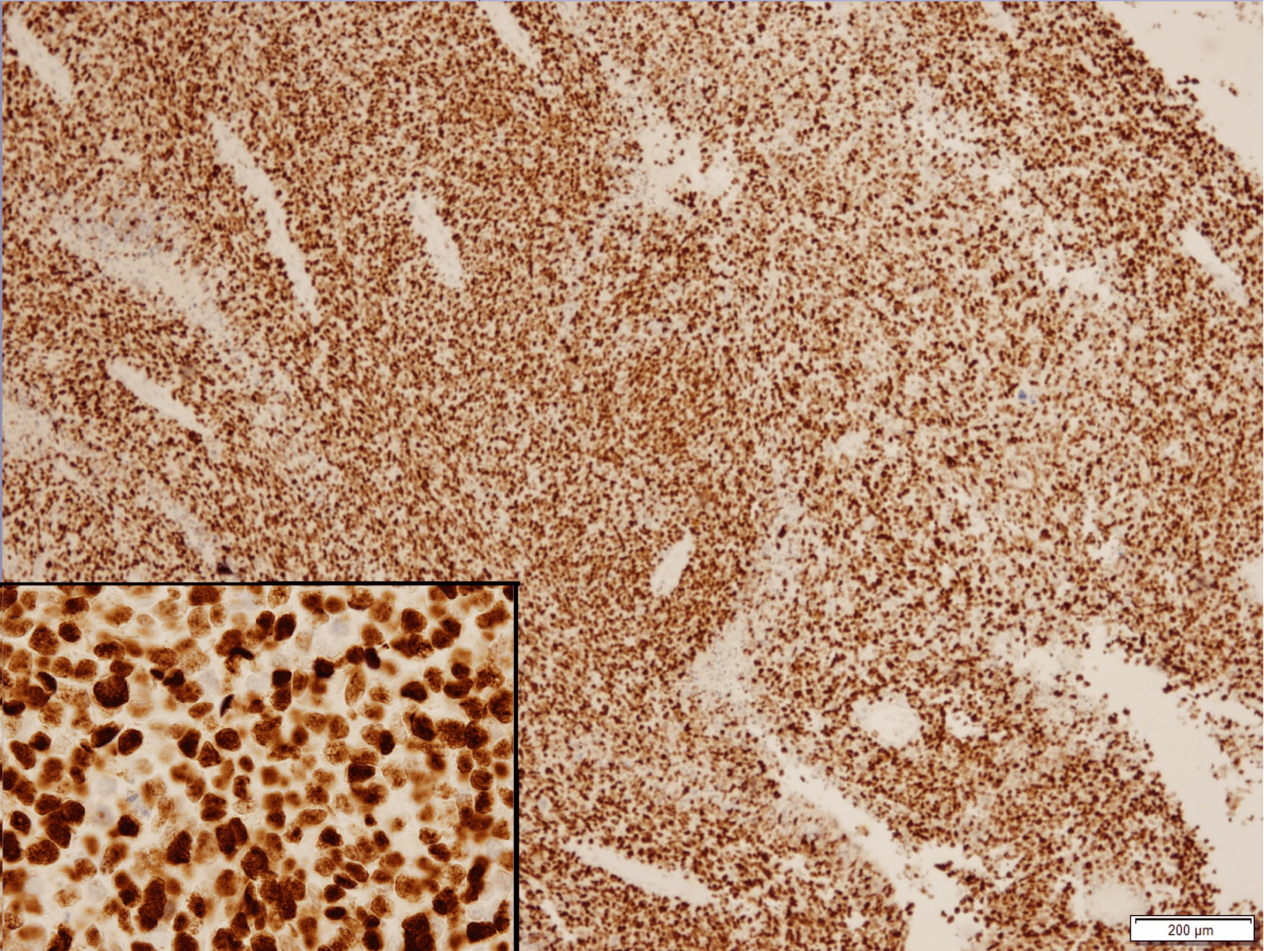
Neoplastic cells show strong and diffuse nuclear positivity for SOX-11 (Immunohistochemical stain, 10x, inset: 40x magnification)

The neoplastic cells were positive for vimentin and CD56 (partial), and negative for S100, HMB45, MART-1, SOX-10, CD3, CD30, CD31, CD45, CD99, AE1/AE3, myogenin, PAX-5 and SMA (See Appendices for detailed information). Positive controls were prepared with confirmed melanocytic lesions positive for all four melanocytic markers, and the sections were placed on the same slides to rule out false negative results in our patient specimen. Both positive and negative controls were stained appropriately. The tumor proliferation index by Ki-67 was 80%. The neoplastic cells were stained partially positive for microphthalmia transcription factor (MiTF) in a small subset of cells (Figure 7) and diffusely positive for SOX-11 (Figure 8).

These findings were diagnostic of malignant neoplasm, suggestive of metastatic melanoma, per the patient’s prior history of intra-parotid melanoma. On histomorphology comparison, the neoplastic cells shared the same histomorphology features with the dedifferentiated focus of prior intra-parotid melanoma. The latter had a loss of staining with SOX-10 and HMB-45. Genomic studies by next-generation sequencing (NGS) (CANCERPLEX®, Kew Inc., Cambridge, Massachusetts, United States) further verified its melanoma origin and revealed somatic mutations involving *NRAS* and telomerase reverse transcriptase (TERT) (c.C-124T promoter variant), loss of *PIK3R1, ATM, CDKN1B, TP53*, and *BRCA1*. No clinically significant mutations were identified including *BRAF, KIT, GNAQ, or BAP1.* No evidence of microsatellite instability was detected, and no human papillomavirus (HPV) types 16/18 or Epstein-Barr virus (EBV) were detected either. The tumor mutation burden was found to be below the mean for melanoma.

## Discussion

We have presented an interesting case of quadruple negative metastatic melanoma with gain of SOX-11 expression and various genetic mutations. Metastatic melanoma is notorious for its ability to imitate a variety of other malignancies. In the absence of a history of melanoma, it is possible to misdiagnose melanoma as other tumors. Because the treatment varies considerably, correct diagnosis is of significant clinical importance for patient management.

Melanomas are well known to express aberrant markers and are also known to display loss of their classic immunohistochemical markers [5-7]. However, loss of all four major melanoma markers is a rare phenomenon and may result in misdiagnosis. Our case of complete loss of all four markers paired with a gain of SOX-11 is unique and presents a diagnostic challenge. SOX-11 is widely expressed in the developing nervous system and is absent in normal adult tissue. Its activation has been shown to block differentiation of mature B-cells into plasma cells in the lymph nodes. Nuclear overexpression of SOX-11 has been identified as a highly specific marker for mantle cell lymphoma (MCL). SOX-11 mRNA and nuclear protein expression are seen in nearly all cyclin D1 positive and negative MCLs, but not in other mature lymphoid neoplasms or in normal lymphocytes [8,9]. Overexpression of SOX-11 can be seen in malignant gliomas and tumors with primitive neuroectodermal differentiation [10]. Reactivity of SOX-11 in melanoma has rarely been reported in the literature before, although neuroectodermal differentiation may be seen occasionally [10-12].

In our case, SOX-11 was found to be overexpressed in metastatic melanoma. Jian et al. suggested SOX 11 expression as a proliferative index of cutaneous malignant melanoma and related with significantly lower survival rate [12]. The role of Sox-11 in metastatic melanoma is still unknown. A battery of immunohistochemical stains was utilized to clarify the primary origin of the tumor. Neoplastic cells were positive for vimentin and partially positive for CD56. All four melanoma markers (S100, HMB45, MART-1, and SOX-10) and hematological markers such as CD3, CD30, CD31, CD45, CD 99, PAX5, and CD138 were negative as well as pan-keratin, myogenin, and SMA. MiTF was reactive focally. Given the patient’s prior history, the splenic mass was diagnosed as the metastasis from previous melanoma, which was confirmed by NGS findings of TERT mutations among others.

In current practice, melanomas are diagnosed with the most sensitive markers SOX-10 and S100, and at least one of the more specific markers such as MART-1 or Melan A, HMB45, and tyrosinase [5,6]. However, it has long been known that melanoma cells can alter their differentiation resulting in loss or gain of several tumor markers [13].

TERT promoter mutations are commonly found in malignant melanomas and recently have been related to advanced stage and decreased survival [14]. Although our patient did not have *BRAF* mutation, which is the most common mutation identified in malignant melanoma [15], he was found to have TERT mutation. This may explain the patient’s advanced disease along with dedifferentiating melanoma metastasis to the spleen.

## Conclusions

Cutaneous melanoma diagnosis is usually a straightforward H&E diagnosis, but occasional cases may present with aberrant phenotypes that mimic a variety of non-melanocytic neoplasms such as sarcomas and create diagnostic confusion. This is especially true for metastatic melanoma because the tumor tends to lose differentiation markers as it progresses. In addition, it is well known that some types of melanoma may not express one or more melanocytic markers such as spindle cell, desmoplastic, and rhabdoid melanomas. Even though the loss of all of the differentiation markers is a rare phenomenon in routine practice, it is crucial to know the “exceptions” to the common patterns of expression. To our knowledge, loss of all four differentiation markers with a gain of SOX-11 expression has not been previously identified in melanomas.

## Appendices

**Table 1:** Institutional antibody clone information

Vimentin	Ventana Roche	Anti-vimentin (V9)	Primary antibody
CD56	Cell marque	Anti-CD56 (MRQ 42)	Rabbit monoclonal antibody
CD99	Ventana Roche	Anti-CD99 (O13)	Mouse monoclonal primary antibody
S100	Ventana Roche	Anti S100	Primary antibody
HMB45	Ventana Roche	Anti melanosome	Mouse monoclonal antibody
Sox 10	Cell marque	Anti Sox10	Rabbit primary antibody
MART1	Ventana Roche	Anti-MART-1/Melan A (A103)	Mouse monoclonal primary antibody
AE1/AE3	Ventana Roche	Anti-pankeratin (AE1/AE3)	Primary antibody
PAX5	Ventana Roche	Anti PAX5 (SP34)	Rabbit monoclonal primary antibody
CD45 (LCA)	Cell marque	Anti CD45 /LCA (2B11 & PD7126)	Mouse monoclonal antibody
CD138	Cell marque	Anti CD138/ syndecan-1 (B-A38)	Mouse monoclonal antibody
